# Editorial

**Published:** 1900-12-15

**Authors:** 


### EDITORIAL.

### "NICE FOR BILL HEADS AND ADVERTISING."

Under the above heading the *Dental Brief* for November quotes an editorial criticism which appeared in the September REGISTER, on what we considered an unwarrented display of business enterprise in professional journalism. The editor of the *Brief* seems to think we had no sufficient cause; as in this instance there was no infraction of the moral code, but simply the exercise of a prerogative wholly without the restraints of morals and obligated only by the more fickle sentimental principles of taste upon which commercial transactions are usually based. Be it so. Does the editor of the *Dental Brief* deny the existence of a fairly well understood and accepted code of ethics of a purely sentimental character, upon which in fact is based all that has any restraining force in our moral or legal codes? In fact, it was the encroachment of this sentimental code which inspired to large extent our comment. It was the unusual and unnecessary effort, so it seemed to us, made to exploit professional *attainments and persons* for commercial purposes which we resented, and against which we had a right to protest, if it were not our duty to do so. Especially since we were asked to co-operate to the extent of an editorial commendation.

To maintain the integrity and dignity of a profession it may be well, even essential, to unite upon a code of morals to which all are expected to subscribe, but the enactment of such a code will not accomplish the object sought unless there is a strong sentiment to uphold and sanction it. But a sentiment so fickle as that suggested by the editorial in question can never supply an ethical code for a great profession which expects to take honorable rank. The dental profession along with its development from a crude calling has developed a strong professional sentiment which is a more significant factor than its formulated code, which is in reality only its outward expression in minimum. And a public action which tends to undermine or threaten the respect for this sentiment, and by so doing hinder its development, should meet with a proper and adequate resentment.

We regret that the editorial in the *Dental Brief* commits both its editors and publishers to the action which we saw fit to criticise, kindly as we thought, by a proceeding which we can no more indorse than we can harmonize their actions about the question at issue. The *publishers* of the DENTAL REGISTER have seen fit to print in its advertising pages, over which they have *absolute control,* some electrotypes to be used by such dentists as do not seem to be able to attract patronage except by resorting to questionable advertising in the public prints and elsewhere. As a matter of "*taste*" our publishers may be justified not only in dealing in such wares but in using their advertising space in the DENTAL REGISTER and the editorial columns of friendly publications, in calling attention to the virtues of these wares. But so far as we are personally concerned these particular cuts are not only exceedingly offensive in themselves, but we greatly regret that there is any occasion for their existence. We have no sympathy with the effort made to supply them to a weak and perverted taste which seems to demand them in sufficient quantity to justify dealers in handling them. The use of such a means of attracting the public notice is degrading to any dentist who will resort to it, and is a very good method of publishing his own incompetency and his lack of appreciation of the proper attitude of a truly professional man; to say nothing of the irreparable injury he is doing to a profession which has conferred upon him its honors, titles and privileges. It is a shame that greed for gain can so possess men that they are willing to sacrifice self-respect and common decency to secure coveted ends, regardless of the rights and privileges of others.

In reply to the editorial inquiry in the *Dental Brief*, we would emphatically state that we *do not* think the cuts in question are "nice for bill heads or advertising." Neither do we think their reproduction in our *editorial columns* necessary to justify our standards of professional methods or morals.

